# Different effects of pesticides on transcripts of the endocrine regulation and energy metabolism in honeybee foragers from different colonies

**DOI:** 10.1038/s41598-023-29257-w

**Published:** 2023-02-03

**Authors:** Verena Christen

**Affiliations:** School of Life Sciences, University of Applied Sciences and Arts Northwestern Switzerland, Hofackerstrasse 30, 4132 Muttenz, Switzerland

**Keywords:** Environmental impact, Biodiversity

## Abstract

Honeybees are important pollinators of many crops and contribute to biological biodiversity. For years, a decline in bee populations has been observed in certain areas. This decline in honeybees is accompanied by a decrease in pollinator services. One factor contributing to the decline of bee colonies is the exposure to pesticides. Pesticide exposure of bees, among other effects, can negatively affect orientation, memory, immune system function and gene expression. Among the altered expressed genes are transcripts of endocrine regulation and oxidative phosphorylation. Endocrine regulation plays an important role in the development of nurse bees into foragers and oxidative phosphorylation is involved in energy metabolism. Most of these transcriptional changes were investigated using mixed aged honeybees derived from the same colony. Experiments using nurse bees or foragers of the same age but from different colonies are rare. In the present study, effects of the two pesticides chlorpyrifos and pyraclostrobin on the expression of transcripts linked to endocrine regulation and oxidative phosphorylation in foragers of the same age from three different colonies are investigated to fill this gap. These two pesticides were selected because negative effects at sublethal concentrations on bees are known and because they are found in pollen and nectar of crops and wild plants. For this purpose, 20–22 days old foragers of three different colonies were exposed to different sublethal concentrations of the selected fungicides for 24 h, followed by analysis of the expression of *buffy*, *vitellogenin*, *hbg-3*, *ilp-1*, *mrjp1*, *2* and *3*, *cox5a*, *cox5b* and *cox17*. Some significant changes in gene expression of both endocrine regulation transcripts and oxidative phosphorylation were shown. Furthermore, it became clear that forager bees from different colonies react differently. This is especially important in relation to the risk analysis of pesticides. In addition, it could be shown that the expression of *hbg-3* in the brain of bees is a robust marker to distinguish nurse bees from foragers at the molecular biological level. In summary, this study clearly shows that pesticides, which are often detected in pollen and nectar, display negative effects at sublethal concentrations on bees and that it is important to use bees from different colonies for risk assessment of pesticides.

## Introduction

Bees are important pollinators of many crops and native plants, contributing about one-third of the human diet globally and providing immeasurable ecosystem services^1^. An estimated 80% of crops in the European Union directly depend upon biotic pollination^2^. Similarly, 80% of all wild plant species depend on insect pollination and 60% show pollination limitation^3^. In addition, bees are also indirectly responsible for the reproduction and maintenance of wild plant communities and biodiversity^4^. These services are so important that a pollinator decline dramatically affects ecosystem services. Indeed, for most regions of the globe and wildest pollinator taxa, no data on whether there have been declines are available. The best estimates are for numbers of domesticated honeybee colonies, which can be obtained for many countries with varying reliability. These suggest that numbers of managed honeybee colonies have decreased in Europe (25% loss of colonies in central Europe between 1985 and 2005)^5^ and markedly in North America (59% loss of colonies between 1947 and 2005)^6^. However, overall global stocks increased by ~ 45% between 1961 and 2008 because of a major increase in numbers of colonies in countries such as China and Argentina^7^. Nevertheless, there are widespread reports of unusually high rates of honeybee colony loss from many parts of the world, sometimes ascribed to a syndrome known as colony collapse disorder (CCD)^8^.

The reasons for bee mortality are many and varied, including habitat loss^5^, parasites and diseases^9,10^ extreme weather^11^ and the exposure to pesticides^12^. Pesticides are biological agents or synthesized substances used for killing or restricting the development of organisms^13^. Pesticides can act against various harmful organisms and are accordingly divided into different categories such as fungicides, herbicides, insecticides, and rodenticides. Among other things, honeybees are exposed to different classes of insecticides including neonicotinoids, pyrethroids and organophosphates^12,14^ and fungicides^15^. The most frequently residues of agrochemicals detected in honeybees are chlorpyrifos, an organophosphate and pyrethroids^15^. For a long time, pesticides have been suspected as one of the main reasons for the decline in bee colonies^16^. By contact with contaminated plants, bees can be exposed to substances that are harmful to them^14^.

The damage pesticides can do to bees includes, but is not limited to, delayed development, impairment of immunity system, shortening the life span of adults^17^, negative effects on visual learning and induced abnormal behavior such as falling, trembling and rapid abnormal movements^18^. The exposure of honeybees to sublethal concentrations of clothianidin had adverse effects on foraging activity and affected dance communication^19^. In a study of Fent and colleagues it was shown that the exposure of honeybees to sublethal concentrations of the pyrethroid cypermethrin altered the expression of transcripts linked to muscular structure, muscular processes, and esterase B1^20^. In addition, effects on transcripts linked to energy metabolism and transcripts coding for detoxification enzymes were detected after exposure of honeybees to different pesticides^21,22^. Furthermore, Fent and colleagues showed adverse effects of pesticides on the expression of transcripts coding endocrine active proteins involved in the transition of nurse bees to foragers^23^.

The study presented here aimed to investigate the effects of the organophosphate chlorpyrifos (CPF) and the fungicide pyraclostrobin (PS) on the expression of selected transcripts in the brain of foragers. For this purpose, foragers of the same age were exposed to sublethal concentrations of CPF and PS for 24 h followed by expression analysis of transcripts involved in oxidative phosphorylation (*cox5a*, *cox5b* and *cox17*) as well as transcripts involved in endocrine functions (*buffy*, *hbg-3*, *ilp-1*, *vitellogenin*, *mrjp1*, *mrjp2* and *mrjp3*) by qPCR. The selection of transcripts is because they regulate very important biological processes such as energy metabolism and conversion of nurse bees to foragers and that previous studies with other pesticides have already shown effects on these transcripts^21–24^. Oxidative phosphorylation plays an important role in energy metabolism and consists of various complexes such as NADH dehydrogenase. The transcripts *cox5a*, *cox5b* and *cox17* studied here encode proteins of the cytochrome C oxidase complex^25^. Therefore, altered expression of these transcripts could be the molecular reason of changed flight behavior and reduced lifetime. The endocrine transcripts analyzed in the present study play an important role in the transition of nurse bees to foragers and the expression levels differ between nurse bees and foragers^26^. Changes in the transcription of these genes may have severe effects on the whole bee colony as the correct ratio of nurse bees and foragers is very important for the whole colony. The two pesticides selected for the study are commonly used in agriculture and some negative effects on bees are already known. Chlorpyrifos, an often-used insecticide in various cultures belongs to the group of triphosphorous organophosphate insecticides and is often applied as formulation. Target organisms are cockroaches, ticks, and fleas^27^. The mode of action is the inhibition of acetylcholine esterase in the brain and the peripheral nervous system upon bioactivation resulting in reduced acetylcholine degradation leading to overstimulation of synapses and therefore neurotoxic effects in target and non-target organisms including honeybees^28^. Some known adverse effects in honeybees are memory and learning disorders. Foraging bees show slowed acquisition and odor generalization. In addition, honeybees have problems with the orientation and therefore difficulties to find the way back to the hive and to flowers leading to less efficient collection of food for the colony^29^. Chlorpyrifos also induces a higher larval mortality rate, and it can be found in nectar, propolis, wax and pollen^30^. Reproduction of honeybee hives is disturbed due to negative effects of chlorpyrifos on development of queens^31^. In a field study, higher mortality rate, reduced foraging for 2–3 days after exposure and reduced floral visitation was observed after CPF exposure^32^. The exposure to sublethal CPF concentrations induced the expression of cytochrome P450 genes and vitellogenin in adult worker bees and decreased the expression of transcripts linked to immune function^33^. In addition, the expression of transcripts linked to the endocrine system was altered after CPF exposure of adult worker bees^23^. The oral LD50 concentration of honeybees is 0.114 mg/bee^34^.

Pyraclostrobin is a strobilurin fungicide whose mechanism of action is the inhibition of mitochondrial respiration, by means of binding to the Qo site of cytochrome b, thereby interrupting fungi energy production^35^. Oral and contact LD50 concentrations of fungicides are normally four orders of magnitude greater than fungicide concentrations detected in honeybee food stores. Therefore, fungicides are regarded as harmless for honeybees^36^. Nevertheless, pyraclostrobin has negative effects on bees. Among other things, pyraclostrobin shows negative effects on the fat body and pericardial cells of foragers^37^, inhibits mitochondrial respiration^38^, reduces the longevity of forager bees^39^, has cytotoxic effects on midgut^39–41^, reduces polysaccharides and midgut proteins^39^ and negatively affects post-embryonic development^40^. In addition, pyraclostrobin is often detected in pollen and nectar of cultivated and wild plant species visited by honeybees^41^.

For the exposure experiments, foragers of the same age were used and a single bee feeding approach was applied because it was shown in a previous study that the data generated in experiments with single bee feeding was less scattered than in experiments with group feeding^24^. Furthermore, foragers of the same age were used to have bees in the same physiological condition and thus to have the most controlled conditions possible. All in all, the experiment was carried out three times with foragers from three different colonies to map the variability between the colonies. In the present study, it was shown that the expression of *hbg-3* in the brain of bees is a good marker to distinguish between nurse bees and foragers, that chlorpyrifos and pyraclostrobin alter the expression of transcripts linked to endocrine regulation and oxidative phosphorylation and that foragers of the same age from different colonies react differently to the same exposure.

## Results

### Expression of transcripts linked to endocrine regulation: intra- and inter-hive variability

**Figure 1:**
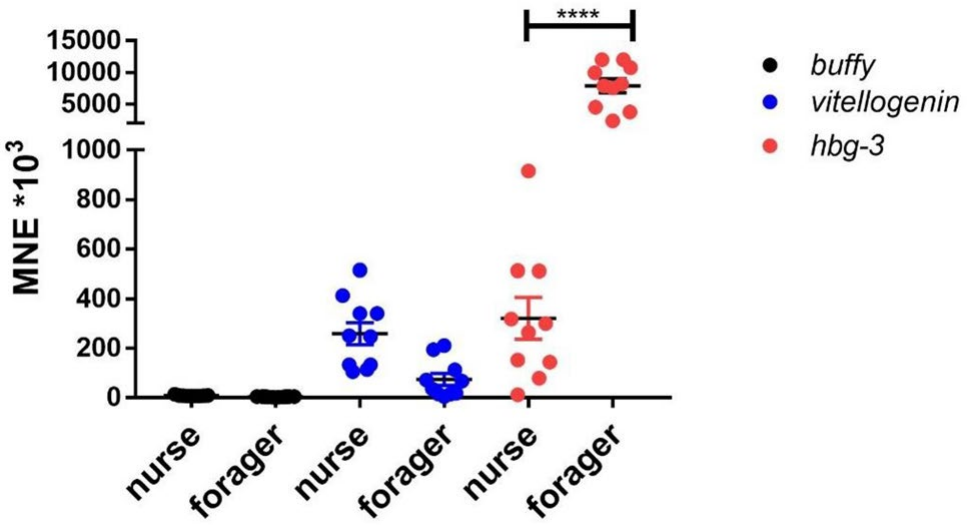
Abundance of transcripts of *buffy* (black symbols), *vitellogenin* (blue symbols) and *hbg-3* (red symbols) in the brain of unexposed nurse bees (n = 10) and foragers (n = 10) from the same hive. Significant differences between nurse bees and foragers with p-value of ≤ 0.05 are marked with *.

**Figure 2:**
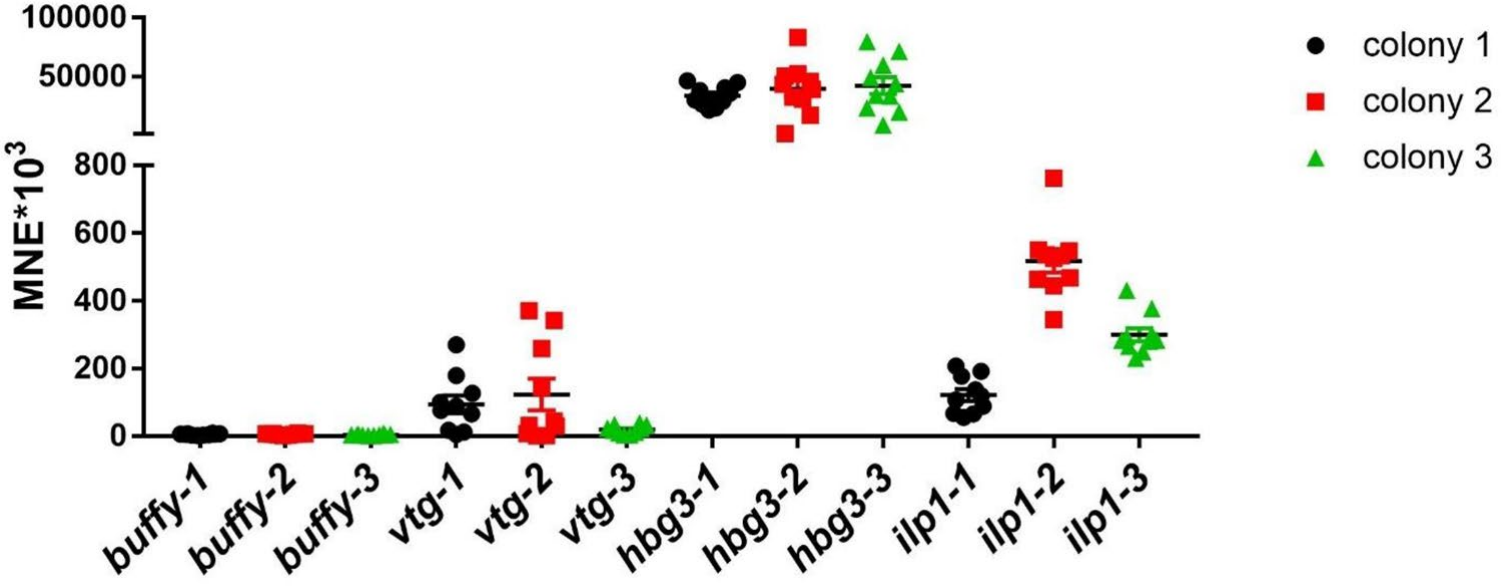
Abundance of transcripts of *buffy*, *vitellogenin*, *hbg-3* and *ilp-1* in the brain of control foragers (n = 10) of colony 1 (black symbols), colony 2 (red symbols) and colony 3 (green symbols). No significant differences were found for the expression of the four examined transcripts between the three different colonies.

**Figure 3:**
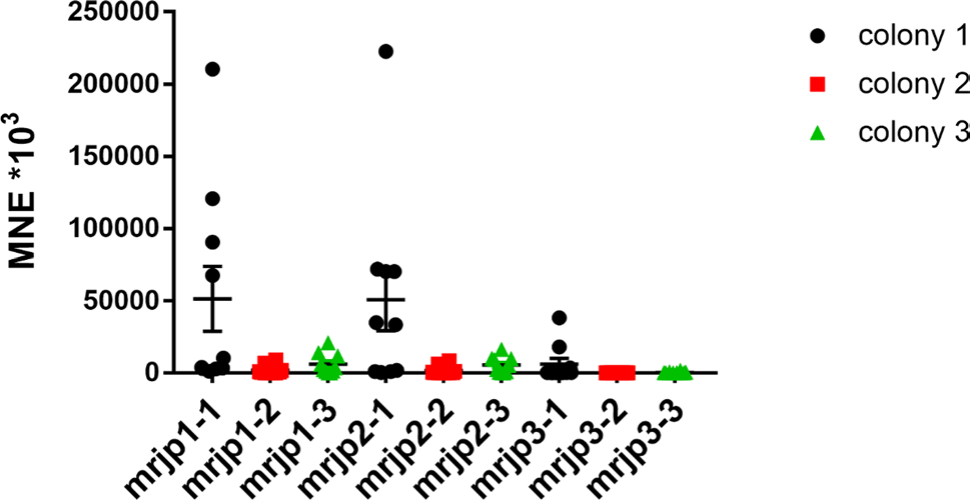
Abundance of transcripts of *mrjp1*, *mrjp2* and *mrjp3* in the brain of control foragers (n = 10) of colony 1 (black symbols), colony 2 (red symbols) and colony 3 (green symbols). No significant differences were found for the expression of the three examined transcripts between the three different colonies.

To investigate the expression levels of the endocrine transcripts *buffy*, *hbg-3* and *vitellogenin*, the expression in the brain of unexposed nurse bees and foragers from the same colony was examined. Of these three endocrine transcripts analyzed, *buffy* showed the lowest expression in both nurse bees and foragers. *Vitellogenin* showed similar expression levels in nurse bees and foragers. *Hbg-3* showed extremely high expression in foragers. The difference in *hbg-3* expression between nurse bees and foragers was significant (Fig. 1). In the next step, the expression levels of the endocrine transcripts were compared between control foragers of the same age from three different colonies. *Buffy* showed the lowest expression with little dispersion, *hbg-3* showed the highest expression. Control foragers from colony 1 and colony 2 showed more scatter for *vitellogenin* than control foragers from colony 3. *Ilp-1* showed lower expression in control foragers from colony 1 compared to control foragers from colonies 2 and 3. However, no significant differences were found in the expression of the four transcripts studied within control foragers from the three different colonies (Fig. 2). In addition to the endocrine transcripts studied so far, the expression of the *major royal jelly proteins 1*, *2* and *3* was also investigated. They showed quite similar expression levels. *Mrjp2* and *3* showed stable expression in all samples examined. In contrast, the expression of *mrjp1* was highly variable, especially in the analyzed control foragers of colonies 1 and 2 (Fig. 3).

### Exposure experiments

**Figure 4:**
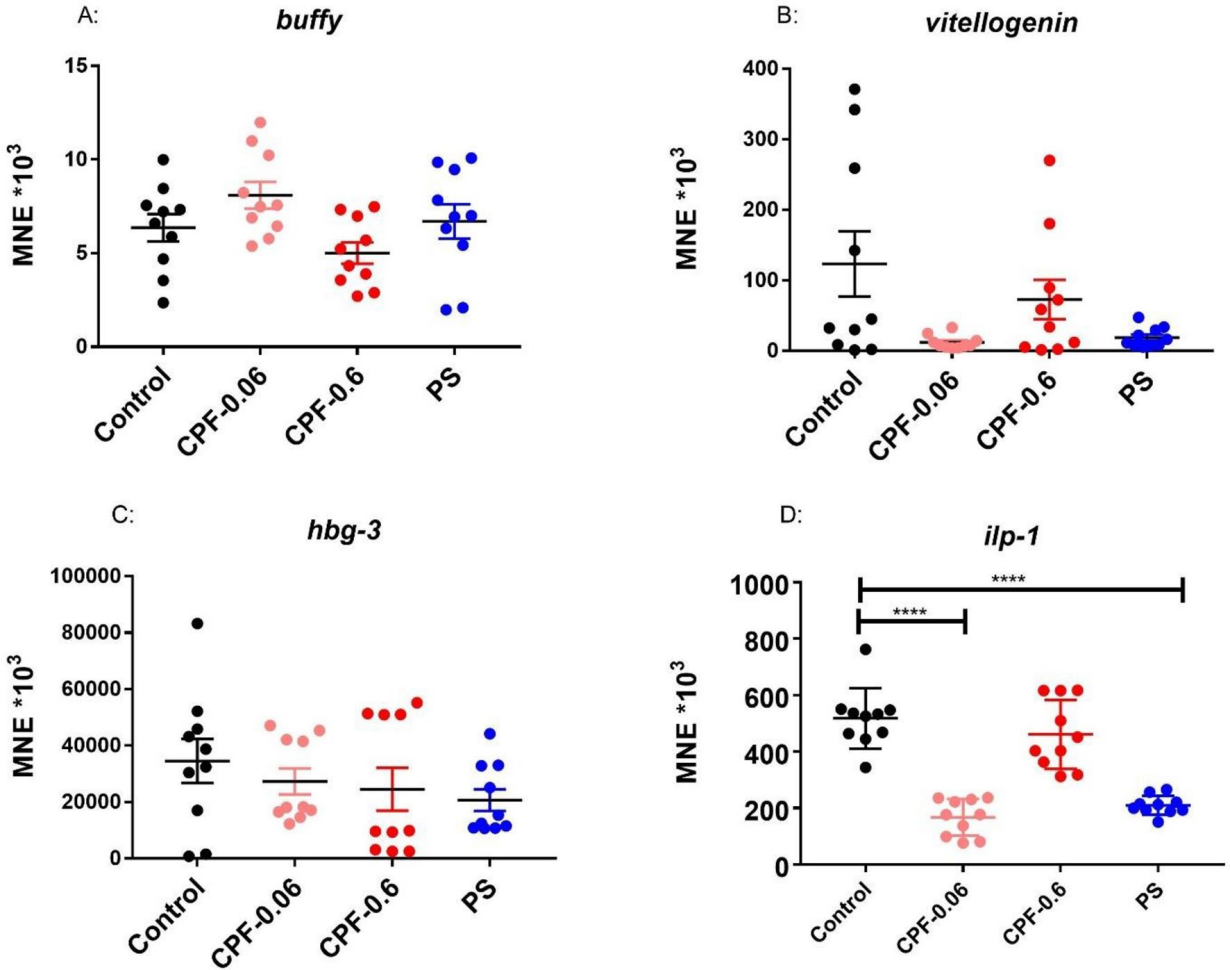
Abundance of endocrine transcripts *buffy* (**A**), *vitellogenin* (**B**), *hbg-3* (**C**) and *ilp-1* (**D**) in foragers of the same age after 24 h exposure to 0.06 and 0.6 ng/bee CPF and 2.65 microg/bee PS, respectively (black dots: controls, light and dark red dots: CPF, blue dots: PS, n = 10). Significant changes are marked with *.

**Figure 5:**
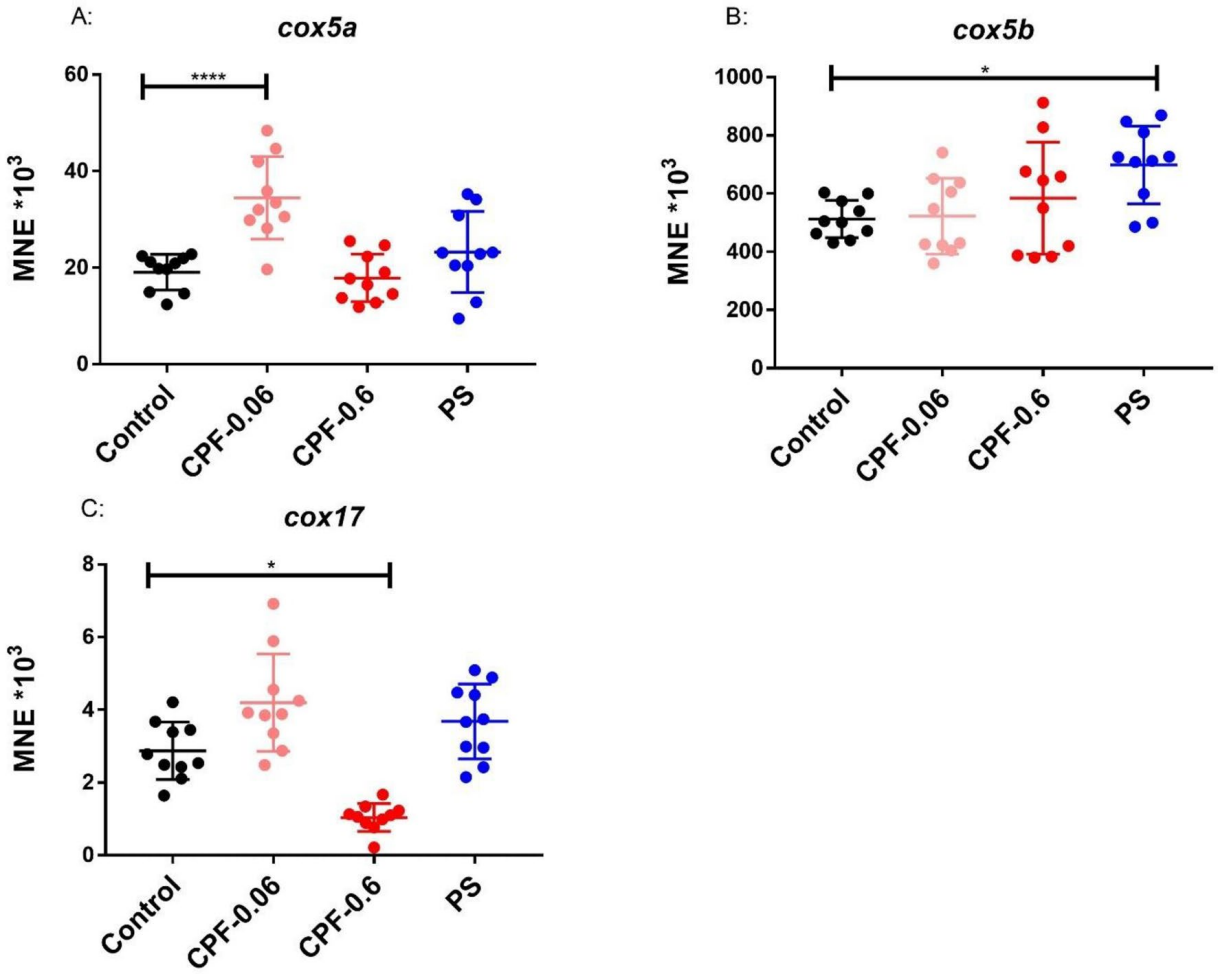
Abundance of *cox5a* (**A**), *cox5b* (**B**) and *cox17* (**C**) in foragers of the same age after 24 h exposure to 0.06 and 0.6 ng/bee CPF and 2.65 microg/bee PS, respectively (black dots: controls, light and dark red dots: CPF, blue dots: PS, n = 10). Significant changes are marked with *.

**Figure 6:**
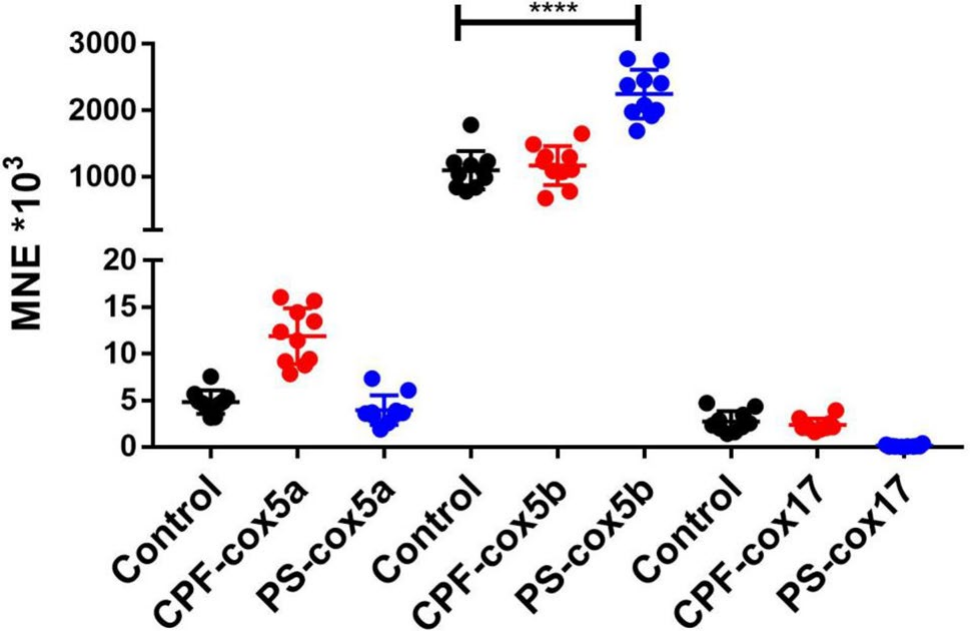
Abundance of transcripts of the oxidative phosphorylation (*cox5a*, *cox5b* and *cox17*) in foragers of the same age after 24 h exposure to 0.6 ng/bee CPF and 2.65 microg/bee PS (black dots: controls, red dots: CPF, blue dots: PS, n = 10). Significant differences between controls and exposed foragers with p-value of ≤ 0.05 are marked with *.

Foragers of the same age from three different colonies were exposed to different concentrations of chlorpyrifos and pyraclostrobin for 24 h, followed by gene expression analysis of endocrine transcripts and transcripts linked to energy metabolism. Due to the limited number of foragers of the same age, only one CPF concentration (0.6 ng/bee) could be tested using foragers of colony 1 and 0.6 ng/bee CPF and 2.65 microg/bee PS could be tested using foragers of colony 3. Both CPF concentrations and PS could be tested using foragers of colony 2. An overview of the experimental setting is shown in Fig. S1. In a first exposure experiment, foragers of the same age from colony 1 were exposed to 0.6 ng/bee CPF for 24 h. Endocrine transcripts as well as transcripts of oxidative phosphorylation (energy metabolism) were analyzed. No significant changes could be detected (Fig. S2). In a second exposure experiment, foragers of the same age from colony 2 were exposed to 0.06 and 0.6 ng/bee CPF and 2.65 microg/bee PS. No significant changes in the expression of *buffy*, *vitellogenin*, *hbg-3*, *mrjp1*, *mrjp2* and *mrjp3* could be detected (Figs. 4 and S3). A significant down-regulation could be shown for *ilp-1* after exposure to 0.06 ng/bee CPF and to PS (Fig. 4). Higher expression levels were shown for *cox5a* after exposure to 0.06 ng/bee CPF and for *cox5b* after PS exposure (Fig. 5). A significant reduction of *cox17* could be detected after exposure to 0.6 ng/bee CPF (Fig. 5). In a third exposure experiment, foragers of the same age from colony 3 were exposed to 0.6 ng/bee CPF and 2.65 microg/bee PS. No significant changes in the expression of *buffy*, *vitellogenin*, *hbg-3*, *ilp-1*, *mrjp1*, *mrjp2*, *mrjp3*, *cox5a* and *cox17* could be detected (Fig. S4). *Cox5b* showed a significant increase after PS exposure (Fig. 6).

## Discussion

In the study presented here, it was shown that the expression of *hbg-3* in the brain of honeybees is a robust marker to distinguish between nurse bees and foragers. In addition, it could be demonstrated that foragers of the same age from different colonies exposed to the same pesticides under controlled conditions react differently and that of the transcripts examined, only *ilp-1* and transcripts linked to oxidative phosphorylation showed altered expression after exposure to chlorpyrifos and pyraclostrobin.

The lifespan of a worker bee is usually 30 to 40 days from spring to autumn^42^, wherein young workers (nurse bees, less than 13 days after eclosion) take care of the brood in the hive by secreting royal jelly, whereas old workers (foragers, more than 18 days) collect nectar and pollen outside the hive^43^. Some physiological changes in certain organs accompany this age-dependent transition in workers. For example, the hypopharyngeal glands (HPGs), a pair of exocrine glands in the worker's head, undergo structural and functional changes associated with this transition. In nurse bees, the HPGs are well developed, synthesize major royal jelly proteins (MRJPs)^44,45^ and express high levels of buffy^44^, whereas in foragers, they shrink and synthesize carbohydrate-metabolizing enzymes that process nectar into honey, such as α-glucosidase III (gene encoding this enzyme: *hbg-3*), α-amylase, and glucose oxidase^44–46^. During aging of honeybees, the expression and enzymatic activity of α-glucosidase III increases between day 18 and day 24^47^. Possible negative effects of pesticides on the transition of nurse bees to foragers may have serious consequences for the whole honeybee colony^29^. Hence, it would be very important in future pesticide risk assessments to analyze the expression of typical nurse bee and forager transcripts in order to detect possible changes in the ratio of nurse bees to foragers in a bee colony at an early stage^29^. But dissection of HPGs from honeybee brains is very difficult and time consuming^48^ and therefore not applicable for analyzing these effects during pesticide risk assessment as it is impossible to analyze many samples in a short time. Therefore, the question arises whether differential expression of *buffy* and *hbg-3* between nurse bees and foragers can also be seen in the whole bee brain. Basically, the expression level of *buffy* in both nurse bees and foragers was very low compared to the other endocrine transcripts. There was no significant difference between nurse bees and foragers (Fig. 1). This could be because by comparing the complete brain tissue, the effects seen in the HPGs alone are diluted and therefore no longer detectable. The expression of *hbg-3* shows clear differences between nurse bees and foragers. In the brain of foragers, the expression level of *hbg-3* is on average a factor of 10 higher than in the brain of nurse bees although the whole brain and not only the HPGs were analyzed (Fig. 1). This clearly shows that the expression of *hbg-3* in the brain of bees is a good and robust marker to distinguish nurse bees from foragers and could be used in future screening assays to detect possible endocrine effects of pesticides on honeybees.

Different responses of bees from different colonies to exposure to pesticides or other stressors have already been shown in other studies. In a study of Yamada 2020, bee colonies were exposed to two neonicotinoids and one organophosphate from October to July of the following year and different parameters such as number of adult bees, number of capped brood cells and intake of pesticides were monitored. For all endpoints analyzed, exposure to the same pesticide showed differences between the bee colonies^49^. One reason for the different reaction of bees to pesticide exposure is the different genetic background. Rinkevich and colleagues found, among other things, different LD50 values for the same pesticide exposure between bees with different genetic backgrounds^50^. In a study by Christen and colleagues, different reactions of different bee colonies to the same exposure were found. This study looked at the homing flight activity and the expression of different marker transcripts after neonicotinoid exposure of three different bee colonies. Both homing flight activity and gene expression differed between the three colonies after neonicotinoid exposure^27^. It has also been shown that bumblebees from different colonies react differently to stress factors. Four different bumble bee colonies were exposed to two stressors, increased temperature and increased CO_2_-levels, and the reaction based on fanning activity was recorded. All four colonies reacted differently to the stressors^51^. In the study presented here, which was carried out under the most controlled conditions possible (bees of the same age, single bee feeding, exposure in the incubator, performance of the individual experiments within three weeks), different reactions were also shown between bees from different colonies. By exposing foragers from colony 1, no significant effects were seen at all, exposure of foragers from colony 3 let only to significant reaction using PS and not CPF. In contrast, exposure of foragers from colony 2 induced several significant responses to both CPF and PS (Table 1). All these results are important for future studies and for future risk assessments of pesticides because experiments with bees from one colony are often carried out in the risk analysis and the possible different sensitivity of bees from different colonies is only little considered.

**Table 1:**
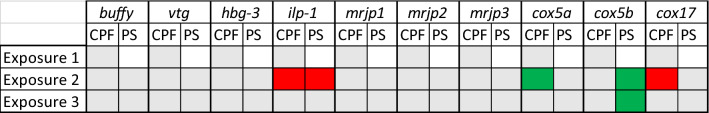
Summary of all transcription data, red: significantly reduced expression, green: significantly increased expression and grey: no change in expression compared to control bees and white: not analyzed.

In the study presented here, gene expression changes of the endocrine-active transcript *ilp-1* and transcripts of oxidative phosphorylation (*cox5a*, *cox5b* and *cox17*) were found (Table 1). Insulin like peptides (ILPs) of invertebrates are important regulators of many steps in life course and functionally homologous to insulin in mammals^52^. *Ilp-1* is expressed in neural and peripheral tissue of honeybees. Its level increases with juvenile hormone and it is transcribed at higher levels in the brain of nutritionally depleted bees and foragers^53^. However, ilp-1 level can drop while foraging continuous, making the link to behavior ambiguous^53,54^. Various pesticides altered the expression of *ilp-1* in the brain of foragers among them thiacloprid, thiamethoxam, cypermethrin, spinosad and chlorothanolin^24–27^. In contrast to the data presented here, which show a downregulation of *ilp-1* after exposure to 0.06 ng/bee CPF, a study by Fent and colleagues showed an induction of *ilp-1* after exposure to 0.6 ng/bee CPF. Exposure to 0.06 ng/bee CPF had no significant effects on *ilp-1* expression in this study^26^. Reasons for this discrepancy could be that in the study of Fent et al. mixed-age foragers were used and therefore the expression of *ilp-1* may already show strong differences within the bees selected for the experiment, even without exposure. In addition, foragers from different colonies may react differently to the same exposure. This phenomenon is also evident in the work presented here. In addition, the experimental setting (group- feeding versus single bee feeding) was different between the study of Fent et al. and the present study here. Due to trophallaxis, the pesticide may have been distributed differently among the exposed worker bees. However, both studies clearly show that CPF can have an influence on the expression of *ilp-1*. Since ilp-1 plays a role in the conversion of nurse bees to foragers^53^, altered ilp-1 levels can have a large effect on the composition of the caste structure in a bee colony. In addition to CPF, also PS inhibited the expression of *ilp-1*. Exposure of foragers from three different colonies to thiamethoxam resulted in an upregulation of *ilp-1* in foragers from two of the three colonies studied^27^. All these data show that pesticides have an impact on endocrine regulation in bees and thus on the transition of nurse bees into foragers. Therefore, it is important to include this endpoint in the risk analysis of pesticides.

The oxidative phosphorylation, a reaction cascade of 5 complexes (I-V), is a key process in energy production from glucose and proteins encoded by *cox5a*, *cox5b* and cox17 belong to the complex IV (cytochrome c oxidase)^28^. Negative effects of pesticides on honeybee energy metabolism are already known. For example, mitochondrial activity and ATP production was altered in the brain and thorax of bees exposed to imidacloprid and fipronil^55^. Furthermore, the fungicide pyraclostrobin displays negative effects on isolated mitochondria of honeybees. Exposure to pyraclostrobin inhibits mitochondrial respiration, reduces mitochondrial membrane potential, and inhibits ATP production^40^. In addition, exposure of bumble bees to different plant protection products had negative effects on mitochondrial energy metabolism in the flight muscles^56^. Moreover, phytochemicals in nectar and pollen can also negatively interact with honeybee energy metabolism^28^. Changed expression of *cox5a*, *cox5b* and *cox17* in mixed-aged foragers after exposure to spinosad also shows the possible effects of pesticides on energy balance in bees^24^. In addition, exposure of mixed-aged foragers to the fungicide chlorothanolin changed the expression of *cox5a*, *cox5b* and *cox17*^25^. Exposure of foragers to the neonicotinoid thiacloprid induced changed transcription of enzymes in-volved in oxidative phosphorylation. These changes were analysed applying RNA sequencing^21^. In a study of Li and colleagues, changed expression of transcripts linked to energy metabolism was detected after exposure of workers to the pyrethroide abamectin^57^. The possible link between disturbed expression of oxidative phosphorylation genes, namely *cox5a*, and thus a disturbed energy balance resulting in altered homing flight behavior of foragers after exposure to neonicotinoids has already been shown^27^. The study here again shows effects of two different plant protection products on the expression of oxidative phosphorylation transcripts (Table 1). Disturbed energy balance can have a variety of negative consequences for bees. Therefore, this endpoint should be included in future risk analyses of pesticides.

In summary, it was shown that the expression of *hbg-3* in the brain of bees can be used as a good marker to distinguish nurse bees from foragers. Furthermore, it was clearly shown that foragers from different colonies react differently to exposure to the same pesticides even when the exposure conditions are as controlled as possible (foragers of the same age, single feeding, experiments conducted within a short period of time). Finally, transcripts of endocrine regulation and oxidative phosphorylation were identified as possible targets of pesticides.

## Materials and methods

### Chemicals

Chlorpyrifos and pyraclostrobin (purities of all > 99%) were purchased from Sigma–Aldrich (Buchs, Switzerland). Stock solutions for each compound were prepared in dimethylsulfoxide (DMSO) and diluted into 20% (w/V) sucrose-solution to a final exposure concentration. Stock and exposure concentrations are summarized in Table 2.

**Table 2:** Information on the exposure experiments and oral LD50 concentrations, *CPF* chlorpyrifos, *PS* pyraclostrobin; both test substances were dissolved in DMSO, DMSO concentration in 20% (w/V) sucrose-solution: 0.1% DMSO, control: 20% (w/V) sucrose-solution with 0.1% DMSO.

Experiment	Bee source	Date of exposure	Oral LD_50_ concentrations (mg/bee)	Exposure concentrations	Concentration test substances/ml 20% sucrose-solution
Exposure 1	Colony 1	20–21.07.2021	CPF: 0.144 mg/bee^37^ PS: > 100 mg/bee (Pesticide Properties Data Base, University of Hertfordshire)	0.6 ng/bee CPF	CPF: 3 and 30 ng/ml PS: 132,5 microg/ml
Exposure 2	Colony 2	22–23.07.2021		0.06 und 0.6 ng/bee CPF, 2.65 µg/bee PS	
Exposure 3	Colony 3	27–28.07. 2021		0.6 ng/bee CPF, 2.65 microg/bee PS	

Selected exposure concentrations were in the non-toxic range.

### Collection of mixed aged nurse bees and pollen foragers

On a day with very good foraging conditions (> 24 °C, sunshine, no wind), brood combs with open brood were pulled out of a Zander colony and the sitting hive bees (approximately 100 bees) were swept with a brush into a large jam jar with sugar dough at the bottom and with air holes in the lid. In parallel, returning pollen foragers (50 bees) were caught directly on the flight board and stored in a large jam jar with sugar dough at the bottom and with air holes in the lid. The bees were then taken to the laboratory and euthanized. The bees were stored at − 20 °C until RNA isolation from the brain.

### Honeybee colonies

The outdoor colonies were situated at a location with no agricultural activity and pesticide use in the Black Forest (Germany, GPS: N 47.7667, E 7.8333). All used bees for the exposure experiment were from three different hives. The colonies had signs of *Varroa destructor* affection and were previously handled with formic acid (August 2020) and oxalic acid (December 2020).

### Exposure experiments

Empty brood combs from miniplus boxes were hung in the brood nest of three Zander colonies (colony 1, 2 and 3). After two days the combs were filled with eggs. As soon as the combs were capped, they were removed from the Zander colonies and hung in empty miniplus boxes. In addition, two food combs were hung in the miniplus boxes. The miniplus boxes were placed next to the Zander colonies with the flight hole closed and daily checks were made for hatched bees. Newly hatched bees were marked with a queen marker (one color per colony) and returned to the original Zander colony. After 20–22 days, the marked bees (foragers) were collected for the exposure experiments. Dates of the exposure experiments can be found in Table 2. In total, empty miniplus brood combs were hung in one Zander colony each on three different days (8.06., 10.06. and 15.06.2021). This means that three colonies were used for three exposure experiments. The collected foragers were transported to the laboratory in a glass with food dough and air holes in the lid. There they were stored for 30 min at 4°. The immobilized bees were distributed into small plastic containers (one bee per container). A pipette tip containing the test substance dissolved in 20% sucrose-solution (20 microl) or solvent as a control was placed in the wall of the containers (Table 2: Exposure concentrations). The containers with the bees were placed in an incubator (25 °C and 60% relative humidity) and regular checks were made to see if the 20 microl of 20% sucrose-solution was taken up. Once the bees had ingested the 20% sucrose-solution (30 min to 1.5 h), they were fed pure 20% sucrose-solution for further exposure (through a 1.5 ml Eppendorf tube with a small hole in the side). In total the bees were exposed for 24 h. In each experiment, 10 bees were exposed to 20% sucrose-solution (controls) and 10 bees to chlorpyrifos and pyraclostrobin, respectively (exposed bees). Thus, there were 10 biological replicates per experiment. Afterwards, the bees were euthanized and stored at -20 °C until RNA isolation.

### Gene expression analysis

The brain of frozen bees was removed in total by opening the cranium using scalpel and forceps. Total RNA of one brain was isolated according to the manufacturer’s instructions (RNeasy mini kit, Qiagen, Hombrechtikon, Switzerland). 1000 ng RNA were reverse transcribed as described before^33^. cDNA was used as a template to perform qPCR using SYBR-Green Fluorescence (FastStart Universal SYBR Green Master, Roche Diagnostics, Basel, Switzerland) and the primer pairs listed in Table 3. Real time PCR amplification was performed on a Bio-Rad CFX96 RealTime PCR Detection System (Bio-Rad, Reinach, Switzerland) under the following conditions: 95 °C for 10 min, followed by 40 cycles at 95 °C for 5 s and 59 °C for 60 s. Melting-curve analyses were performed after each run to ensure the formation of specific products. Analysis of the raw data was done as described by Simon-Delso et al.^58^. In detail, quantification of transcripts was made using the relative quantification method and normalized to the housekeeping gene *ribosomal protein S5* (*rpS5*, this selection is based on the stable transcription of *rpS5* across seasons in a previous study^59^) with Qgene method (http://www.qgene.org/qgene/index.php)^58^ according to the equation:

$${\text{MNE}} = \frac{{E_{{{\text{ref}}}} \wedge {\text{Ct}}_{{\text{ref:mean}}} }}{{E_{{{\text{target}}}} \wedge {\text{Ct}}_{{\text{target:mean}}} }}$$

**Table 3:** Sequences and qPCR efficiency of analyzed transcripts.

Primer	Forward	Reverse	qPCR efficiency	Reference
*rps5*	AATTATTTGGTCGCTGGAATTG	TAACGTCCAGCAGAATGTGGTA	1.94	^60^
*buffy*	CATGGCACTTCTCATCCTTTT	GAGAACGGTTTCAGCATCAAT	2.07	^44^
*vitellogenin*	GCAGAATACATGGACGGTGT	GAACAGTCTTCGGAAGCTTG	1.95	^61^
*hbg-3*	TACCTGGCTTCGTGTCAAC	ATCTTCGGTTTCCCTAGAGAATG	2.06	^44^
*ilp-1*	GCTCAGGCCTGTGCTCGAAAAGT	CGTTGTATCCACGACCCTTGC	1.94	^54^
*mrjp-1*	CACAGCCCAAGATGGAATTT	AAGAAGACGCCACTCTTTGA	2.02	^22^
*mrjp-2*	GGAAAGGGAGGGCTAGTCTC	TCGATCGTCATTTTGGCATA	2.01	
*mrjp-3*	ATTGCCGTAAACGCCACTAC	CAATCGATGGAAGGAATCGT	2.01	
*cox5a*	TCGCATGATGGACCACAAGA	AGGTACAAGATCCAGCCGC	2.03	^24^
*cox5b*	TGGATGTGGTTACATGATGGC	AAAGTGGTGCAACTTGAGTAAG	1.95	
*cox17*	AACCTTGTTGTGCTTGT	ATGTGCTTCTATTAAATCCC	2.26	

where MNE stands for mean normalized expression; E_ref_ is housekeeping gene efficiency; E_target_ is target gene efficiency; Ct_ref;mean_ is mean Ct value for housekeeping gene; and Ct_target;mean_ stands for mean Ct value for target gene. Data are presented as the transcriptional expression of the gene of interest relative to that of the housekeeping gene multiplied by the factor 10^3^ for better visualization. The efficiency of each primer pair was assessed by making a dilution series of honeybee cDNA, which was used to perform qPCR as described above. The efficiency was calculated using Qgene (https://www.gene-quantification.de/eff.html). The efficiencies of the different primer pairs are shown in Table 3. Alterations of mRNA abundance in chlorpyrifos and pyraclostrobin exposed brain samples were always compared to solvent control (DMSO) samples to determine the effects of plant protection products.

### Data processing and statistical analysis

GraphPad Prism software was used to perform statistical analysis and to draw graphics. Differences between treatments were assessed by one-way ANOVA followed by Sidak’s multiple comparisons test. Results of transcripts were given as means ± standard error of means. Differences were considered statistically significant and marked with one asterisk at 0.05 > p > 0.01, or two asterisks at 0.01 > p > 0.001 or three asterisks at 0.001 > p > 0.0001. ANOVA and Sidak’s summary tables of all significant data are shown in the Supplementary material.

## Supplementary Information

Supplementary Information 1.

Supplementary Information 2.

## Data Availability

All data generated or analysed during this study are included in this published article (and its supplementary information files).
