# Supplementary material for: Different effects of pesticides on transcripts of the endocrine regulation and energy metabolism in honeybee foragers from different colonies
Source: Sci Rep. 2023 Feb 3;13:1985. doi: 10.1038/s41598-023-29257-w (PMC9898565; doi:10.1038/s41598-023-29257-w)
Supplement: Supplementary file 1 — Supplementary Information 1. [file 41598_2023_29257_MOESM1_ESM.docx]

Different effects of pesticides on transcripts of the endocrine regulation and energy metabolism in honeybee foragers from different colonies

Verena Christen^1^

^1^ University of Applied Sciences and Arts Northwestern Switzerland, School of Life Sciences, Hofacker-strasse 30, CH-4132, Muttenz, Switzerland.

**Running title:** Gene expression analysis after pesticide exposure

Summary tables (Tables S1-S4) of ANOVA and Sidak`s test of all significant data

Fig. S1: Graphical presentation of the experiments carried out

Fig. S2: Abundance of endocrine transcripts and transcripts of the oxidative phosphorylation in foragers of experiment 1

Fig. S3: Abundance of endocrine transcripts in foragers of experiment 2

Fig. S4: Abundance of endocrine transcripts in foragers of experiment 3

Table S1


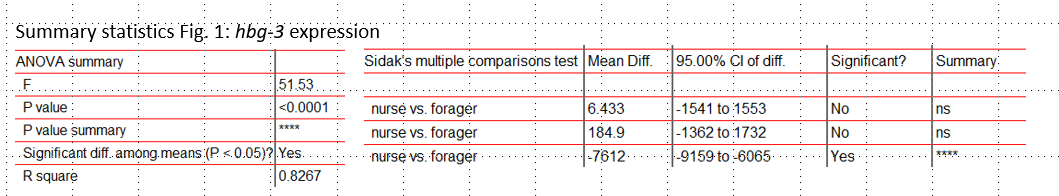


Table S2


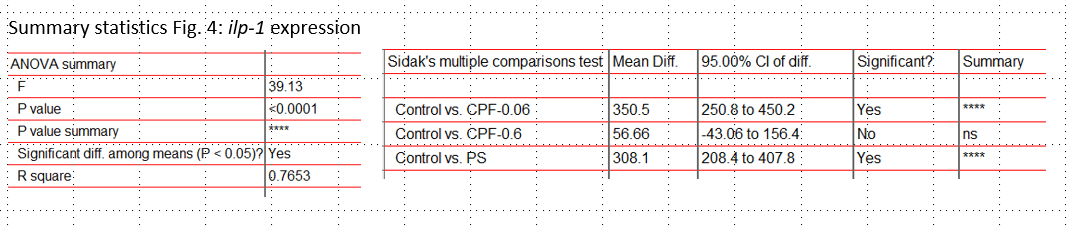


Table S3


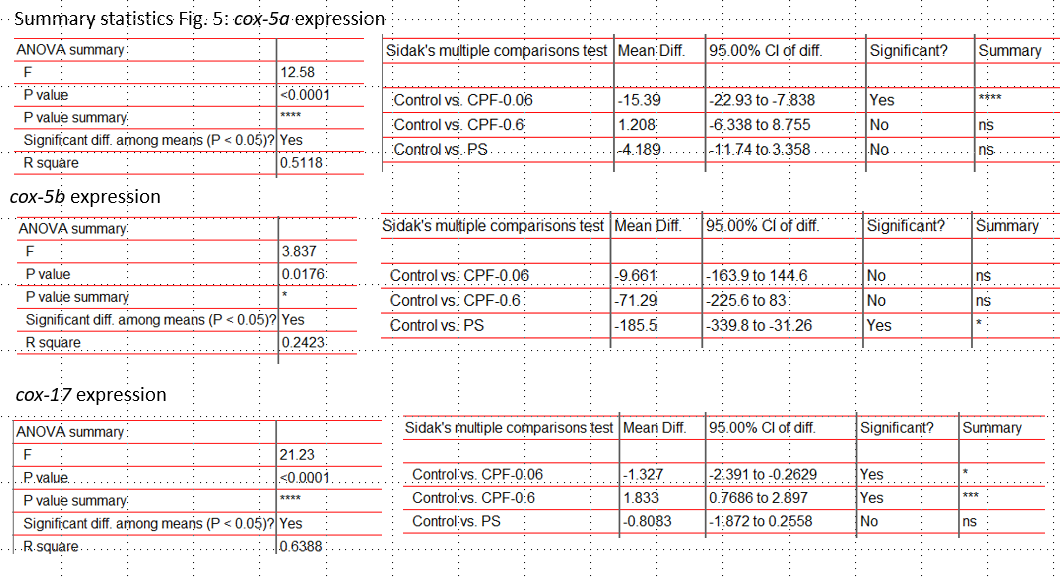


Table S4


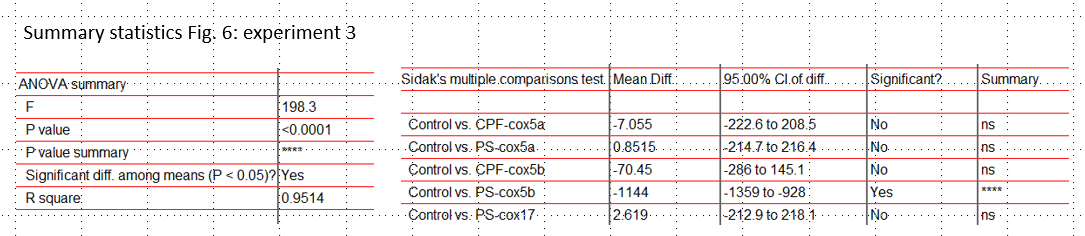


Fig. S1:


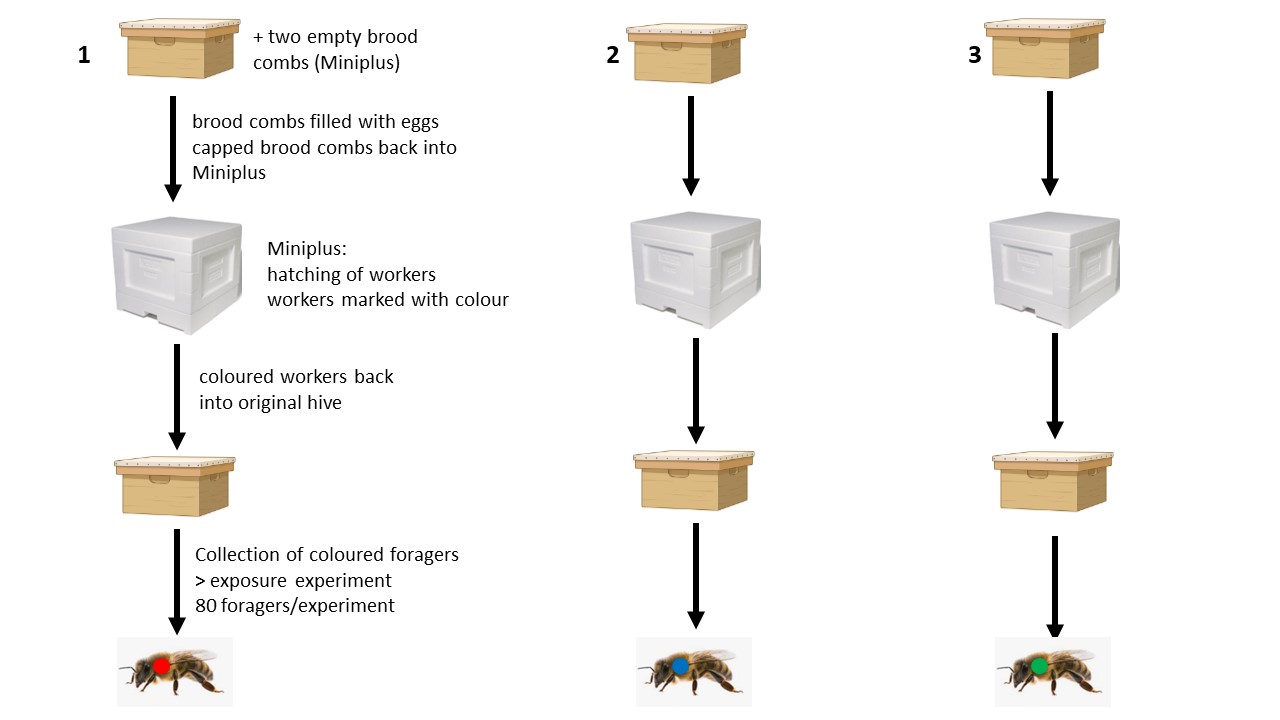


Figure S1: Experimental design: Forager bees of the same age were bred in three different bee colonies. For each colony (1, 2 and 3), the newly hatched bees were marked with a different color. Details about the procedure are described in the methods section.

Fig. S2:

Figure S2: Abundance of endocrine transcripts (*buffy*, *vitellogenin*, *hbg-3*, *ilp-1*, *mrjp1*, *mrjp2* and *mrjp3*) and transcripts of oxidative phosphorylation (*cox5a*, *cox5b* and *cox17*) in foragers of the same age after 24h exposure to 0.6 ng/bee chlorpyrifos (black dots: controls, red dots: CPF, n=10). No significant changes were detected.

Fig. S3:


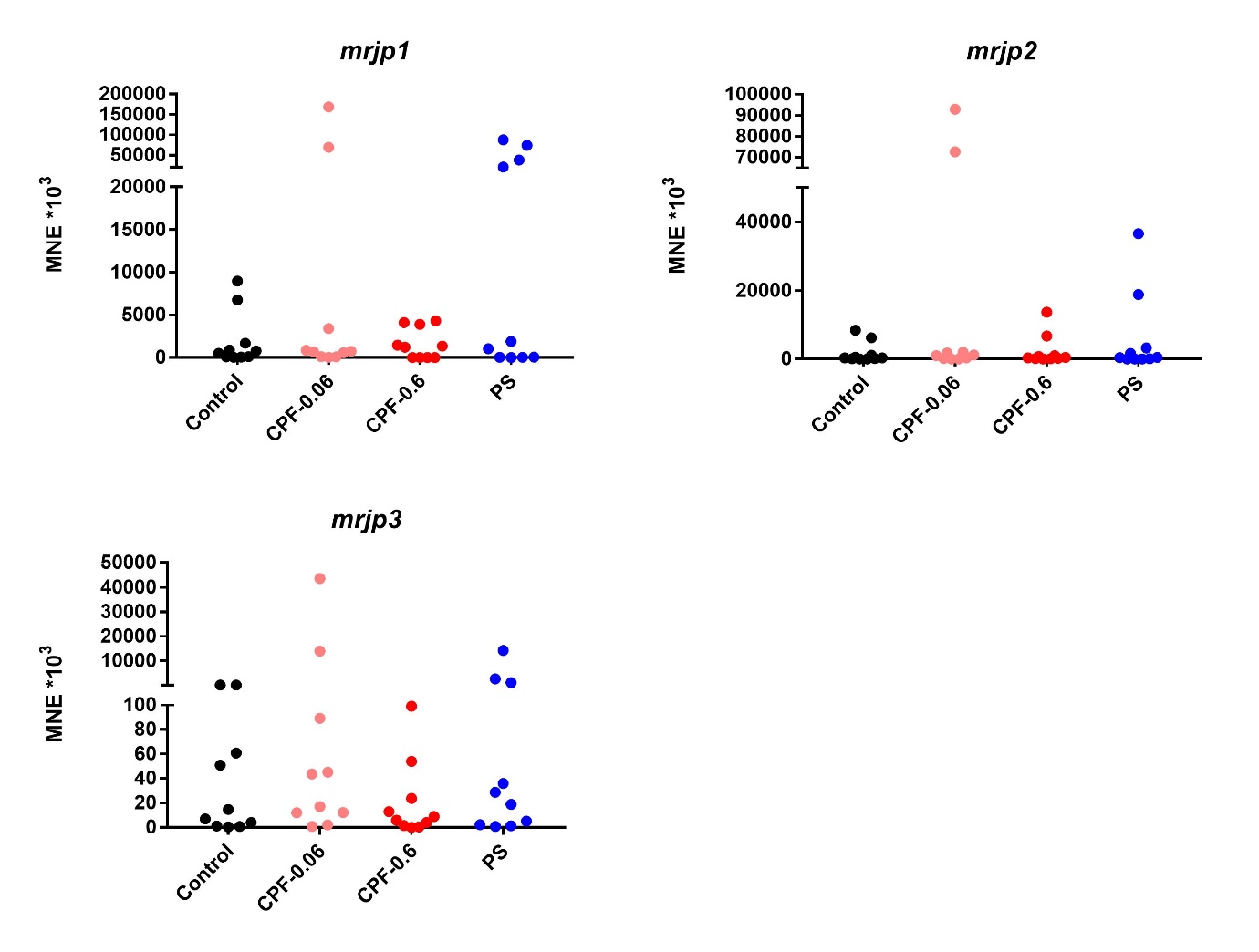


Fig. S3: Abundance of endocrine transcripts *mrjp1*, *mrjp2* and *mrjp3* in foragers of the same age after 24h exposure to 0.06 and 0.6 ng/bee CPS and 2.65 g/bee PS, respectively (black dots: controls, light and dark red dots: CPF, blue dots: PS, n=10). No significant changes were found.

Fig. S4


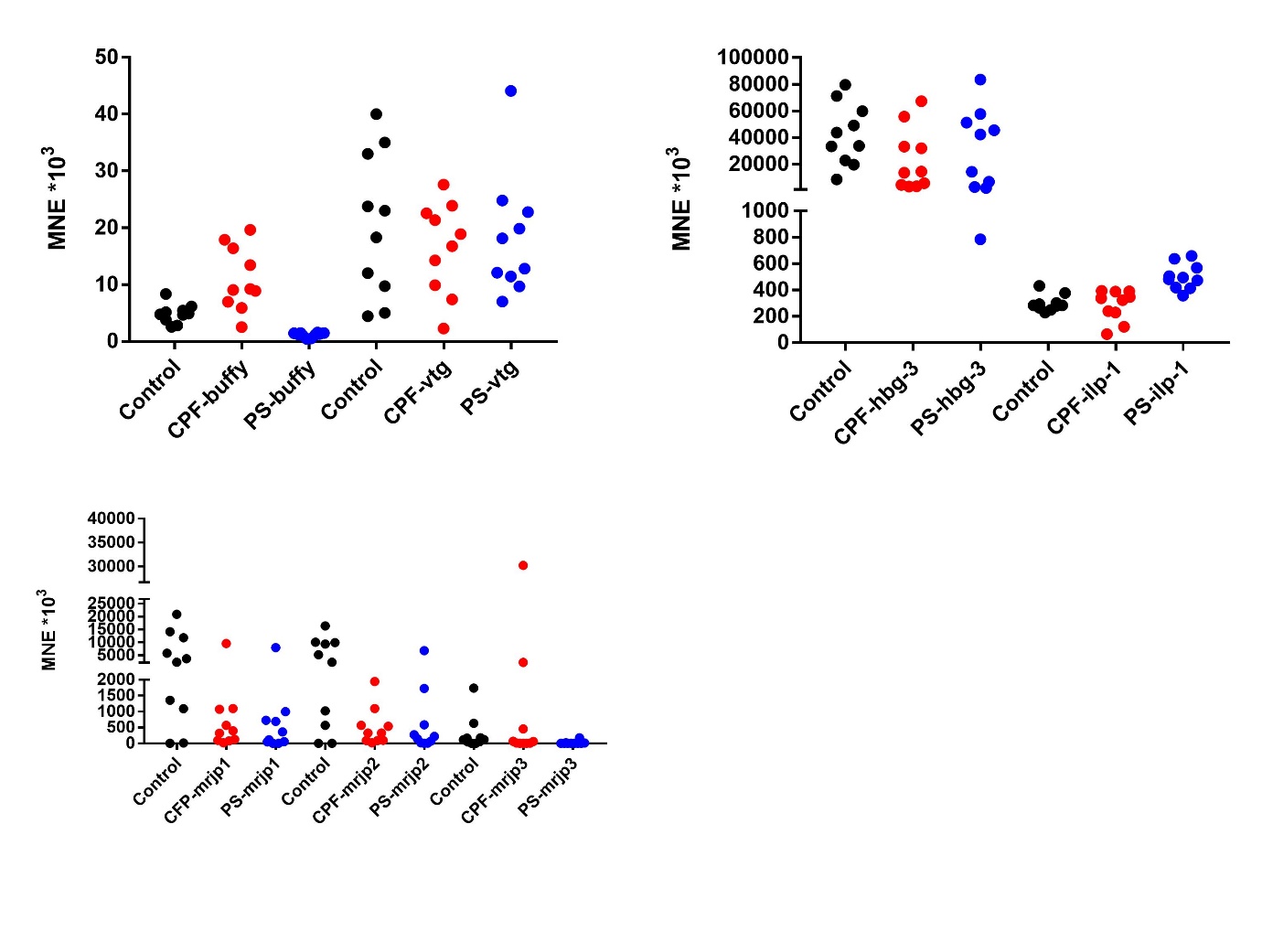


Figure S4: Abundance of endocrine transcripts (*buffy*, *vitellogenin*, *hbg-3*, *ilp-1*, *mrjp1*, *mrjp2* and *mrjp3*) in foragers of the same age after 24h exposure to 0.6 ng/bee CPF and 2.65 mg/bee PS (black dots: controls, red dots: CPF, blue dots: PS, n=10). No significant changes were detected.
